# Clinical and Radiological Evaluation of Turmeric Powder as a Pulpotomy Medicament in Primary Teeth: An *in vivo* Study

**DOI:** 10.5005/jp-journals-10005-1404

**Published:** 2017-02-27

**Authors:** Rajiv N Purohit, Manohar Bhatt, Kanchan Purohit, Jitendra Acharya, Rajesh Kumar, Rakesh Garg

**Affiliations:** 1 Assistant Professor, Department of Dentistry, Sardar Patel Medical College Bikaner, Rajasthan, India; 2 Principal and Head, Department of Pediatric and Preventive Dentistry, Jaipur Dental College, Jaipur, Rajasthan, India; 3 Dental Surgeon, Aashirwad Nursing Home, Bikaner, Rajasthan, India; 4 Senior Demonstrator, Department of Dentistry, Sardar Patel Medical College Bikaner, Rajasthan, India; 5 Associate Professor, Department of Prosthodontics, Jaipur Dental College, Jaipur Rajasthan, India; 6 Senior Resident, Department of Dentistry, Sardar Patel Medical College Bikaner, Rajasthan, India

**Keywords:** Anti-inflammatory, Herbal product, Pain, Primary tooth, Turmeric.

## Abstract

Many plants with biological and antimicrobiological properties have been studied since there has been a relevant increase in the incidence of antibiotic overuse and misuse. In dentistry, phytomedicines have been used as anti-inflammatory, antibiotic, analgesic, and sedative agents. Turmeric is used extensively in foods for its flavor and color, as well as having a long tradition of use in the Chinese and Ayurvedic systems of medicine, particularly as an anti-inflammatory and for the treatment of flatulence, jaundice, menstrual difficulties, hematuria, hemorrhage, and colic. This article studies ahead with a new idea of using powdered turmeric as a material of pulpotomy procedure in primary teeth.

**How to cite this article:**

Purohit RN, Bhatt M, Purohit K, Acharya J, Kumar R, Garg R. Clinical and Radiological Evaluation of Turmeric Powder as a Pulpotomy Medicament in Primary Teeth: An *in vivo* Study. Int J Clin Pediatr Dent 2017;10(1):37-40.

## INTRODUCTION

Primary teeth are the best space maintainer. Therefore, it is important to maintain the primary dentition in dental arch, provided it can be restored to function and remain disease free. Dental diseases involving pulp and periapi-cal tissues can be hyperemia (reversible/irreversible), pulpitis (acute/chronic ulcerative/chronic hyperplastic), pulp degeneration (calcific/fibrous/atrophic/ internal resorption), and necrosis. These diseases can be treated by indirect pulp-capping therapy, direct pulp-capping, and pulpotomy or pulpectomy procedures depending on the extent of involvement of the pulp.^1^

The primary goal of endodontic treatment in primary teeth is to eliminate infection and to retain the tooth in a functional state until their normal exfoliation time, without endangering the permanent dentition or the general health of the child. The use of biocompatible substances has become a major interest in modern dentistry, especially when direct contact with the dental tissues is necessary. In this sense, the field of phytotherapy, which is the use of plants or plant extracts for medicinal purposes, has experienced a remarkable advance in recent years. This has stimulated the investigation of different herbal products with potential therapeutic properties for dental applications. Phytomedicines, simply defined, are area-specific category of plant drugs.^1^

Earlier, till today, several materials have been investigated as pulp-capping materials, such as calcium hydroxide, mineral trioxide aggregate, formocresol, ferric sulfate, enamel matrix derivative, propolis, *Copaifera langsdorffii* oleoresin, recombinant human bone morphogenetic protein 7, lasers, and aloe vera gel.^2^

Herbal products have been used since ancient times in folk medicine, involving both Eastern and Western medicinal traditions. Many plants with biological and antimicrobiological properties have been studied since there has been a relevant increase in the incidence of antibiotic overuse and misuse. In dentistry, phytomedicines has been used as anti-inflammatory, antibiotic, analgesic, and sedative agents. In endodontics, because of the cytotoxic reactions, most of the commercial intracanal medicaments used and their inability to eliminate bacteria from dentinal tubules, trend of recent medicine attends to use biologic medication extracted from natural plants.^3^

*Curcuma longa,* a perennial herb and member of the Zingiberaceae (ginger) family, grows to a height of 3 to 5 feet and is cultivated extensively in India, China, and other countries with a tropical climate. It has oblong, pointed leaves and funnel-shaped yellow flowers. The rhizome, the portion of the plant used medicinally, is usually boiled, cleaned, and dried, yielding a yellow powder. Turmeric is used extensively in foods for its flavor and color, as well as having a long tradition of use in the Chinese and Ayurvedic systems of medicine, particularly as an anti-inflammatory and for the treatment of flatulence, jaundice, menstrual difficulties, hematuria, hemorrhage, and colic.^4^

The aim this study to evaluate clinically and radio-graphically turmeric powder as a pulpotomy material evaluated at different time intervals of 3 weeks, 2, 4, and 6 months.

## MATERIALS AND METHODS

The present study shows a total of 50 children aged between 4 and 9 years, who were having carious unilateral or bilateral deciduous molar in maxillary and mandibular arch, were selected from the outpatient clinic of Department of Pedodontics with Preventive Dentistry, Jaipur Dental College and Hospital, Jaipur, India. Out of screened 50 children, 15 were selected. They were in good general health having no history of systemic illness or hospitalization. After selection of patient, institutional ethical committee clearance was obtained from concerned authorities, and evaluation of constituents and safety of the sample is undertaken by Guru Nanak Institute of Pharmacy, Hyderabad, Andhra Pradesh, India. The parents or guardians of the child were informed about the condition of their child’s dentition. Once the patients were selected, the teeth for the study that were selected had following clinical and radiographic criteria.

### Preparation of Turmeric Powder and Radiolucent Material

The dried rhizomes of turmeric were grounded to fine powder under hygienic conditions to form a turmeric powder. This turmeric powder, distilled water, and radiolucent material were mixed on a glass slab with the help of stainless steel spatula, and mixing ratio of turmeric powder, distilled water, and radiolucent material was 1:3:3.

### Methodology

**Fig. 1:**
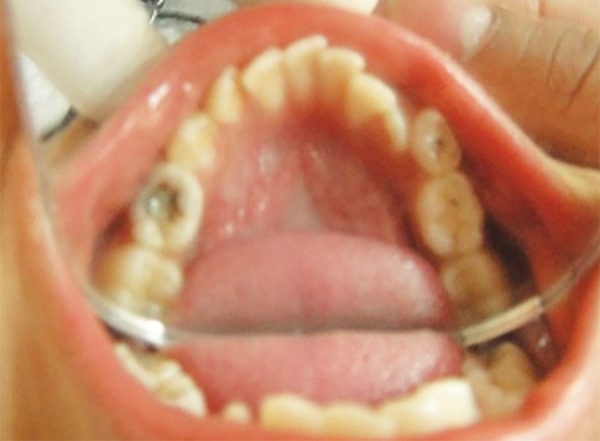
Carious tooth (85)

Local anesthesia for the selected tooth to be treated if required was achieved by desired nerve block, using 2% lignocaine hydrochloride with adrenaline 1:80000 followed by isolation was achieved using rubber dam, and the access cavity (Fig. 1) was done depending on the extent of lesion. The carious dentin was excavated with the help of spoon excavator and large round bur followed by roof of the pulp chamber removed, making sure that overhanging edges were eliminated and a proper access to root canal orifice was achieved.

**Fig. 2:**
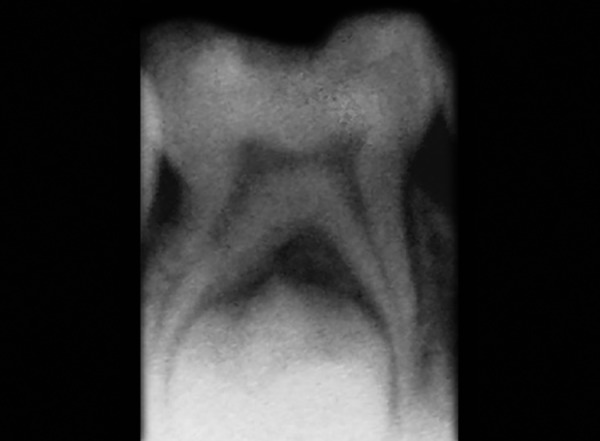
Preoperative radiograph of 85

To arrest the bleeding pulp stumps, a cotton pellet soaked in distilled water was used for 1 to 2 minutes, and then the pulp stumps was fixed with the mixture of turmeric powder (Fig. 2), distilled water, and radiopaque material with the help of cement carrier. Followed by a series of restoration, i.e., a layer of zinc oxide eugenol, glass ionomer cement restoration, and composite restoration, oral hygiene instructions were given.

Patients were recalled and teeth with above-mentioned mixture were evaluated in 3 weeks, 2, 4, and 6 months follow-up.

On evaluation visits, the treated teeth were evaluated for pain, tenderness to percussion, presence of mobility, and signs of pathology or fistula, whereas radiographic examination included any presence of radiolucency in periapical or furcal area.

**Fig. 3:**
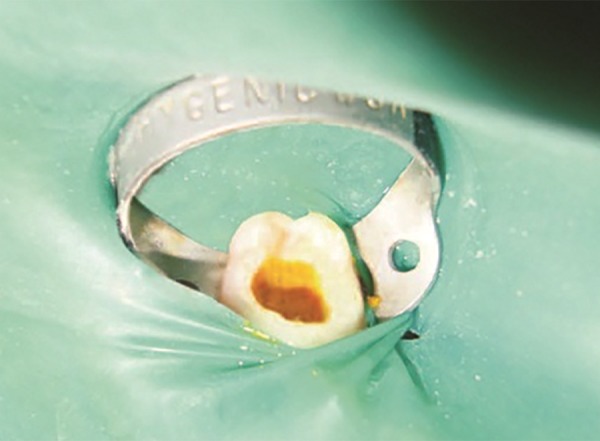
Turmeric placed in 85

**Fig. 4:**
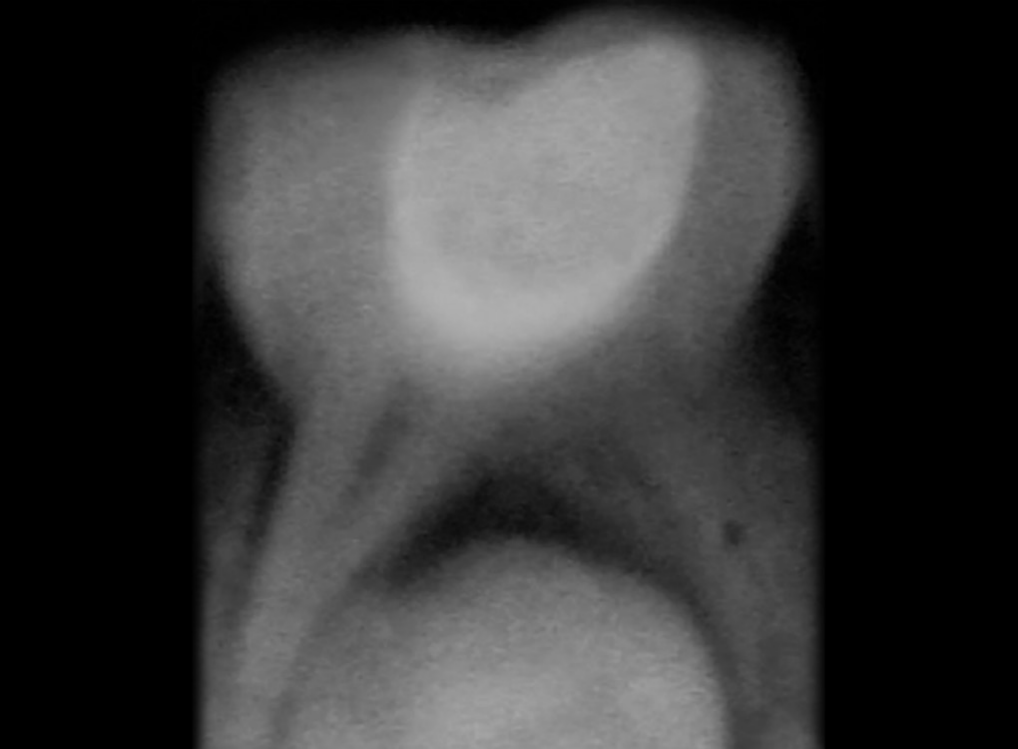
Postoperative radiograph after 6 months of 85

The cases treated were considered as successful when there is absence of pain, tenderness on percussion, mobility, and signs of pathology or fistula. The case was regarded as a failure when one or more of the following signs were present: Internal root resorption, furcation radiolucency, periapical bone destruction, pain, swelling, sinus/fistula, or tenderness on percussion (Fig. 3). The cases were considered successful radiographically when there are no signs of radiolucency during postoperative visits (Fig. 4).

**Table 1:** **Table 1:** Distribution of various clinical signs and symptoms reported from baseline and after treatment (in %)

*Interval*		*Pain*		*Tenderness*		*Mobility*		*Sinus and* *fistula*	
Baseline		15 (100.00)		0 (0.00)		0 (0.00)		0 (0.00)	
After 3 weeks		0 (0.00)		0 (0.00)		0 (0.00)		0 (0.00)	
After 2 months		0 (0.00)		0 (0.00)		0 (0.00)		0 (0.00)	
After 4 months		0 (0.00)		0 (0.00)		0 (0.00)		0 (0.00)	
After 6 months		1 (6.66)		0 (0.00)		0 (0.00)		0 (0.00)	

**Table 2:** **Table 2:** Distribution of presence and decrease of radiolucency in furcation at various intervals

*Interval*		*Presence of radiolucency* * in furcation area*	
Baseline		0 (0.00)	
After 3 weeks		0 (0.00)	
After 2 months		0 (0.00)	
After 4 months		0 (0.00)	
After 6 months		0 (0.00)	
Remark			

## RESULTS

The present study shows the observation after 3 weeks, 2, 4, and 6 months follow-up. There was only one patient (6.66%) who reported pain after 6 months, and none of the patients reported tenderness, mobility, and sinus/ fistula after 6 months (Table 1). Radiographically, no changes were seen after 3 weeks, 2, 4, and 6 months in furcal area (Table 2).

## DISCUSSION

Pulp medicaments should induce the regeneration of the remaining pulp tissue, and any potential inflammatory response caused by their application must not cause harm to the pulp. Based on these prerogatives, the present study evaluated the use of turmeric powder as pulp medicaments, since these substances are considered to be biocompatible and present therapeutic properties that are widespread in folk medicine and supported by research-based evidence.

The present study showed that none of the patients reported with tenderness, mobility, and sinus/fistula after 6 months. This reduction was highly significant: 1 patient (6.66%) out of 15 primary molars reported with pain, 6 months after the treatment, which could be because of restoration failure. Thus, the end of 6-month reduction of pain in 93.34% of cases was 100%. Radiographically, no changes were seen after 3 weeks, 2, 4, and 6 months in furcal area. The study by Chandra and Gupta^5^ suggested curcumin as a potent anti-inflammatory with specific lipoxygenase and cyclooxygenase 2-inhibiting properties. Studies on animals, as well as *in vitro* and *in vivo* studies, demonstrate turmeric’s effectiveness of decreasing both acute and chronic inflammation, and Chainani-Wu^6^ also found, in a phase I human trial with 25 subjects using up to 8,000 mg per day for 3 months, no toxicity from curumin. Five other human trials using 1,125 to 2,500 mg per day have also found it to be safe.

## CONCLUSION

Pulpotomy treatment using turmeric powder in primary teeth has shown good clinical and radiographic success. However, we advocate further studies with histologic evaluation to attain the further efficacy and healing effects of turmeric on dental pulp.
